# *COVID-19 Stats:* COVID-19 Incidence,* by Urban-Rural Classification^†^ — United States, January 22–October 31, 2020^§^

**DOI:** 10.15585/mmwr.mm6946a6

**Published:** 2020-11-20

**Figure:**
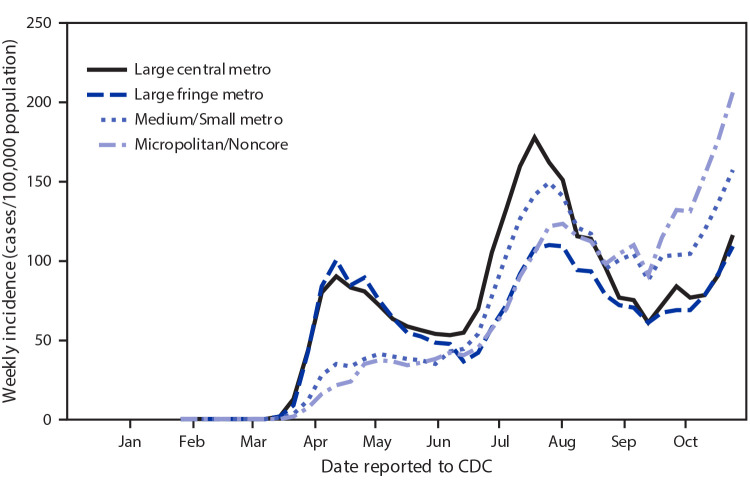
Early in the pandemic, from mid-March to mid-May, COVID-19 incidence was highest among residents of large central and large fringe metropolitan areas. Beginning in mid-April, incidence in large metropolitan (central and fringe) areas declined and then increased similarly among all urban-rural areas. In September 2020, COVID-19 incidence sharply increased, and it remains highest among residents of medium/small metropolitan areas and micropolitan/noncore areas, indicating increased spread into rural communities. In October, weekly incidence was increasing steadily among all urban-rural areas.

**Source:** Protect USAFacts. https://usafacts.org/visualizations/coronavirus-covid-19-spread-map/.

